# Pilot Study: Exploring the Feasibility of Individual Voluntary Waiting Period Settings Using Postpartum Recovery Indicators in Dairy Cows

**DOI:** 10.3390/ani15223331

**Published:** 2025-11-19

**Authors:** Younghye Ro, Jiyeon Kim, Eunwoo Chun, Eunhui Choe, Eunsong Lee, Woojae Choi, Danil Kim

**Affiliations:** 1 Department of Large Animal Medicine, College of Veterinary Medicine, Kangwon National University, Chuncheon 24341, Republic of Korea; dura82@kangwon.ac.kr; 2 Department of Farm Animal Medicine, College of Veterinary Medicine, Seoul National University, Seoul 08826, Republic of Korea; melilsa8037@snu.ac.kr (J.K.); alisonchun@snu.ac.kr (E.C.); 3 Farm Animal Clinical Training Research Center, Institutes of Green-Bio Science and Technology, Seoul National University, Pyeongchang 25354, Republic of Korea; choieh08@snu.ac.kr; 4 Department of Theriogenology, College of Veterinary Medicine, Kangwon National University, Chuncheon 24341, Republic of Korea; eslee@kangwon.ac.kr; 5 Department of Large Animal Medicine, College of Veterinary Medicine, Gyeongsang National University, Jinju 52828, Republic of Korea; 6 Research Institute for Veterinary Science, Seoul National University, Seoul 08826, Republic of Korea

**Keywords:** cattle, calving, voluntary waiting period, farm productivity, dairy

## Abstract

This study aimed to identify hematological markers that can objectively assess postpartum recovery in dairy cows, which is essential for determining an individualized voluntary waiting period (VWP). Twenty Holstein cows were divided into early recovery and late recovery groups based on their clinical uterine recovery. Blood samples were collected from coccygeal vessels and analyzed for inflammatory and metabolic markers, including serum amyloid A (SAA), haptoglobin (HPT), cortisol (COR), substance P (SP), interleukin-6 (IL-6), total cholesterol (T-Chol), beta-hydroxybutyrate, and non-esterified fatty acids (NEFA). Both SAA and HPT significantly decreased after recovery compared to immediately after parturition (*p* < 0.001), whereas T-Chol and NEFA significantly increased and decreased over time, respectively, indicating recovery from a negative energy balance status. No significant changes were observed in COR, SP, or IL-6 levels. Thus, SAA and HPT are sensitive indicators of postpartum inflammation, whereas T-Chol and NEFA levels reflect metabolic recovery. Using these hematological markers enables a more objective and customized setting of VWP for each cow, potentially improving reproductive efficiency and farm profitability. Further studies are warranted to establish cutoff values for each parity and validate the applicability of individualized VWP models.

## Simple Summary

Following calving, dairy cows necessitate a sufficient recovery period before the next pregnancy because lactation and calving cause a negative energy balance and inflammation, respectively. This recovery period, known as the voluntary waiting period (VWP), has traditionally been set uniformly for all cows, although recovery rates differ by case. Here, we compared cows that recovered quickly after calving with those that recovered more slowly to identify blood markers reflecting recovery status. Blood parameters related to inflammation and energy metabolism were analyzed. The levels of two acute-phase proteins, serum amyloid A and haptoglobin, were high immediately after calving but decreased as recovery progressed, indicating their potential as markers of uterine and systemic recovery. Over time, total cholesterol increased, whereas beta-hydroxybutyrate and non-esterified fatty acids decreased, suggesting improved energy balance. Other stress and immune marker levels demonstrated no significant changes. Therefore, blood analysis can provide an objective method for evaluating postpartum recovery instead of relying solely on clinical or subjective assessments. Establishing individualized VWPs based on physiological recovery may reduce unnecessary waiting periods, improve conception rates, and enhance overall farm productivity.

## 1. Introduction

Reproductive performance, along with mastitis incidence, is among the most important productivity indicators on dairy farms [1,2,3,4,5,6,7]. In the livestock industry, economic profit is heavily dependent on good reproductive performance, and the reproductive cycle is affected by nutritional status, stress, and biological rhythms [1,3,4,7,8,9]. Factors influencing reproductive performance include calving intervals (CIs), conception rates at first insemination after delivery, and the number of inseminations required for pregnancy; ultimately, shortening the days open after parturition is paramount [1,3]. Therefore, postpartum reproductive recovery is critical in dairy farms [1,3,5,7]. The factor that influences days open is the voluntary waiting period (VWP), which varies from a minimum of 40 days to a maximum of 230 days, depending on the farm environment and management [5,10,11,12]. To improve farm profitability, the goal time for the first artificial insemination (AI) after calving is generally 50–70 days; nonetheless, this does not reflect individual recovery differences [5]. Minimizing the VWP can be beneficial to farm productivity when considering milk yield, costs, and management efforts [13,14]. Generally, cows with high milk yields recover slowly from reproductive recovery after calving; additionally, an average economic loss of €22.38/cow occurs when the VWP is extended from 9 to 15 weeks [9,11,14,15]. Nevertheless, excessive VWP shortening may increase the number of inseminations for pregnancy or expose the cow to other diseases due to incomplete recovery following shortened CIs [10,11,16]. Recently, increasing VWP beyond the traditional standard in high-yielding dairy cows has been shown to increase productivity in the next parity [10,11,16]. Furthermore, excessive VWP shortening despite slow uterine recovery attributable to poor body condition, metritis, uterine trauma at calving, and peripartu-rient metabolic disorders may cause losses to the farm, such as decreased conception rate and increased AIs for pregnancy [14].

Importantly, postpartum dairy cows must fully recover their overall health, including their reproductive system, to prepare for their next pregnancy [6]. This includes involution of the uterus, which has been enlarged due to pregnancy and parturition, to its normal size and elimination of intrauterine inflammation [17,18,19]. Methods for assessing inflammatory recovery include blood markers and cytobrushing of the endometrium to count polymorphonuclear leukocytes [6,7,20,21,22]. Furthermore, lactation begins immediately after parturition, demands substantial energy, and challenges immune and metabolic function [23]. Accordingly, complete resolution of postpartum inflammation and recovery from the transition-period negative energy balance (NEB) are essential for improving reproductive efficiency [20,23].

Most dairy farms set goals for their reproductive indices and make various efforts to achieve them [24,25], including evaluating the uterus after parturition, as well as systemic condition assessments and blood tests to determine the optimal conditions for re-fertilization after parturition [24,25]. Specifically, several recent studies have evaluated various factors that may reduce reproductive efficiency, even in the absence of visible clinical symptoms, including acute phase protein (APP) or stress indicators after parturition [23,24,26]. In cattle, serum amyloid A (SAA) and haptoglobin (HPT) are widely used APPs, and cortisol (COR) and substance P (SP) have been studied as markers of inflammation and stress [2,24,27,28,29,30,31,32,33]. Additionally, studies on cytokines, such as interleukin (IL)-1β, IL-6, and tumor necrosis factor-alpha (TNF-α), have been conducted in humans for a long time as well as recently in cattle [4,17,18,19,34].

Among the several APPs, SAA is mainly used in cattle, increases dramatically in inflammatory diseases, and acts as a sensitive inflammation marker [24,27,28,31,35]. SAA is synthesized in the liver and is a useful indicator of the condition of the reproductive system [25,27]. Moreover, it can screen for uterine infections before calving [25,30]. Besides SAA, HPT has been used as an inflammation biomarker [23,28,30,31,33,35]; specifically, it tends to increase 10–100-fold under inflammatory conditions [36,37]. HPT is secreted by hepatocytes in response to inflammation and is associated with postpartum diseases, fertility, and milk production [28,36,37,38].

As a glucocorticoid, COR affects immune responses and induces APP production [29,33,39,40]. It is used as the main stress marker along with SAA and HPT [29,33,37,39,40]. In other species, such as dogs and humans, CRP is used as a marker of stress and inflammation; nevertheless, in cattle, CRP is not often used as a biomarker [24,34,40]. Recently, several studies have used SP as a stress biomarker, which has previously been used as a pain-specific biomarker [38,40,41,42]. For the activation of T and B cells and initiation of the acute phase reaction (APR), cytokines, including IL-6, are secreted from macrophages and neutrophils [3,18,19,31,34,43]. Importantly, IL-6 is mainly used as a prognostic marker rather than as a diagnostic biomarker [17,43].

In dairy cows, the recovery rate after calving and the timing of the transition to breeding can affect subsequent reproductive performance, particularly the VWP, significantly impacting the economic profits of farms. Therefore, rapid post-calving recovery and accurate assessment before return to reproductive function are key strategies for improving farm productivity. This study aimed (i) to compare and analyze the hematological markers related to inflammation and energy status in groups with clinically rapid and slow post-calving recovery and (ii) to identify objective hematological markers that can be used to determine individual VWP.

## 2. Materials and Methods

### 2.1. Animals and Group Definitions

Twenty Holstein dairy cows (average parity, 1.6) from the Seoul National University Farm were used in this study. A skilled veterinarian performed rectal examinations of postpartum cows at 1-week intervals to assess the condition of the uterus and ovaries. Cows presenting excessive lochial discharge within the first week postpartum underwent uterine lavage with a 1% povidone–iodine saline solution. By 5 weeks postpartum, no cows had detectable intrauterine contents on examination, and group allocation was therefore based solely on uterine recovery status at that time. The recovered cows were returned to breeding program. Based on the timing of clinical uterine recovery, cows were classified into two groups; after sampling commenced, animals were enrolled sequentially until each group reached n = 10. The early recovery (ER) group included cows that, by 5 weeks postpartum, had completed uterine involution with normal elasticity and showed resumption of ovarian cyclicity on ultrasonography, evidenced by a dominant follicle. The late recovery (LR) group comprised cows that, at 5 weeks postpartum, had not returned to prepartum uterine size, remained flaccid, or failed to show resumption of ovarian cyclicity. For ER and LR cows, complete clinical recovery was achieved on average 30.3 days (±4.6) and 51.5 days (±8.5) after calving, respectively. Information for each sampling period and group is presented in Table 1.

### 2.2. Blood Samples and Analyses

Blood samples were collected from all calving cows at weekly intervals using EDTA tubes (K_2_EDTA, BD Vacutainer, BD, Franklin Lakes, NJ, USA) and heparin tubes (Lithium heparin, BD Vacutainer, BD, Franklin Lakes, NJ, USA) from the coccygeal vessel. All samples were transported under refrigerated conditions, and a complete blood count (Scil Vet Abc Plus, HORIBA, Kyoto, Japan) was conducted with EDTA samples immediately after arrival at the laboratory. Heparinized samples were centrifuged at 3000 rpm for 15 min, and the plasma was separated and stored below −70 °C until plasma chemistry and APP analyses. Hematological analysis was performed only on blood samples collected immediately after delivery (period 1), at ER (period 2), and at LR (period 3).

Plasma chemistry (Cobas C311, Roche, Indianapolis, IN, USA) was performed to determine lipid metabolism and energy balance status.

To assess inflammation and stress status, five parameters were measured using the enzyme-linked immunosorbent assay (ELISA): SAA (Phase Serum Amyloid A Multispecies ELISA, Tridelta Development Ltd., Kildare, Ireland), HPT (Bovine Haptoglobin ELISA kit, ICL Inc., New York, NY, USA), COR (Cortisol ELISA, TECAN Group Ltd., Männedorf, Switzerland), SP (Substance P ELISA kit, Enzo Biochem, Inc., Farmingdale, NY, USA), and IL-6 (Mouse IL-6 Uncoated ELISA Kit, Thermo Fisher Scientific Inc., Waltham, MA, USA).

### 2.3. Statistical Analyses

To analyze changes over time within groups, a one-way repeated-measures analysis of variance with the Tukey method was performed, and Student’s *t*-test was conducted for the same time period between groups. All statistical analyses were performed using SigmaPlot 15 software (GraphPad LLC., San Diego, CA, USA).

## 3. Results

The values for all measured parameters are presented in Supplementary Table S1.

### 3.1. Inflammatory State

**Figure 1:**
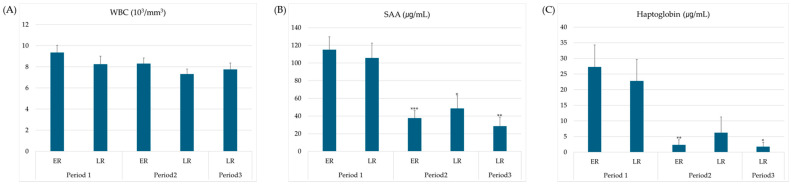
Changes in blood inflammatory parameters in each group and period. Data are expressed as means ± standard error. Significant differences from period 1 at each subsequent period are indicated (*, *p* < 0.01; **, *p* < 0.01; ***, *p* < 0.001). (**A**) White blood cells count in whole blood; (**B**) plasma concentration of serum amyloid A; (**C**) plasma concentration of haptoglobin. WBC, white blood cell; SAA, serum amyloid A; ER, early recovery; LR, late recovery.

WBC count, SAA, and HPT changes over time, which may indicate an inflammatory state, are shown in Figure 1. There were no significant differences in WBC counts between the groups or periods. The SAA values in the ER group were significantly lower in the ER2 than in ER1 (*p* < 0.001). Furthermore, the values significantly decreased in LR2 and LR3 compared to those in LR1 (*p* < 0.05, *p* < 0.01, respectively). Similarly, HPT levels exhibited a significant decrease over time in each group compared with values immediately after delivery (*p* < 0.05). Specifically, a significant decrease was observed in the ER group during period 2 (*p* < 0.01), whereas in the LR group, compared with period 1, no significant difference and a significant decrease were observed in periods 2 and 3, respectively (*p* < 0.05).

### 3.2. Acute-Phase Response

**Figure 2:**
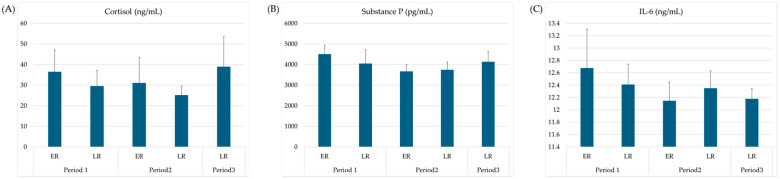
Changes in stress or pain markers and cytokines in each group and period. Data are expressed as means ± standard error. (**A**) Cortisol plasma levels; (**B**) plasma concentration of substance P; (**C**) interleukin-6 plasma levels. IL-6, interleukin-6; ER, early recovery; LR, late recovery.

IL-6, a cytokine that induces an acute phase response, as well as cortisol and substance P, demonstrated no significant differences across the periods or groups (Figure 2C, Figure 2A, and Figure 2B, respectively).

### 3.3. Feed Intake and Energy Balance

**Figure 3:**
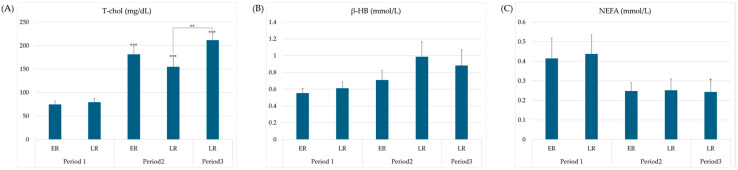
Changes in blood parameters for energy metabolism in each group and period. Data are expressed as means ± standard error. Significant differences from period 1 at each corresponding period are indicated (*, *p* < 0.01; **, *p* < 0.01; ***, *p* < 0.001). (**A**) Plasma concentration of total cholesterol; (**B**) Plasma concentration of beta-hydroxybutyrate in plasma; (**C**) Plasma concentration of non-esterified fatty acid (NEFA). T-chol, total cholesterol; β-HB, beta-hydroxybutyrate; NEFA, non-esterified fatty acid; ER, early recovery; LR, late recovery.

Serum chemistry, which reflects the recovery of feed intake and energy balance in animals, showed significantly increased T-Chol levels over time in both groups after parturition (Figure 3C, *p* < 0.001); in the LR group, significant differences were observed between periods 2 and 3 (Figure 3C, *p* < 0.01). No significant differences were observed in beta-hydroxybutyrate (β-HB) according to the time and groups, while non-esterified fatty acid (NEFA) levels were significantly lower in LR3 than in LR1 (*p* < 0.01; Figure 3B and Figure 3C, respectively).

## 4. Discussion

Previous studies have attempted to shorten the VWP to improve farm productivity [44]; nevertheless, recently, the need to set individual VWPs has emerged [12]. Specifically, while achieving a target VWP does not cause major issues for primiparous cows, a standardized VWP goal may lead to decreased productivity in subsequent parities in multiparous cows, especially those with high milk yields [14,15,16]. Notably, dairy cows are at risk of many diseases during the peripartal period, representing a significant proportion of mortality and culling [1,3,5,7,14]. Some dairy farms intentionally extend the CI to reduce these risks [10,12]. However, extending the CI also prolongs the VWP, thereby increasing the body condition score of cows and causing fattening, potentially augmenting the risk of disease in subsequent parities [10]. According to Burgers et al., if VWP is excessively extended from 50 to 200 days, the levels of β-HB and NEFA after parturition significantly increase in subsequent parities, increasing the risk of metabolic disease occurrence [10,20]. Conversely, Proudfoot et al. reported that, although extending the VWP delays the first insemination after parturition, it can reduce the number of inseminations required for pregnancy from 1.9 to 1.6 by allowing sufficient uterine and energy recovery [4]. Considering the results of various studies, applying VWP equally to all dairy cows, regardless of their recovery status, may result in unexpected economic losses. Therefore, applying an individualized VWP after evaluating each cow may be most helpful for farm productivity.

In cattle, APP can increase because of various causes, including inflammation, transport, stress, and vaccination [27,31,35]. An APP that augments during APR is classified as positive; representative examples include SAA, HPT, and CPR [35]. Contrastingly, transferrin and albumin, which decrease during the APR, are considered to be negative [35]. SAA, a representative positive APP, demonstrated a tendency to increase significantly in the first week after parturition [35,45,46]. Here, SAA and HPT levels were high in ER1 and LR1 in the first week after parturition, and tended to decrease significantly over time. In our previous study on APP analysis according to foot-and-mouth disease and lumpy skin disease vaccination, the SAA level was below 50 μg/mL in normal conditions, increasing to over 100 μg/mL after vaccination [31]. Here, the level of SAA similarly increased to over 100 μg/mL immediately post-delivery, subsequently decreasing to under 40 μg/mL post-recovery. According to current evidence, SAA increases up to 1000-fold during the acute phase of disease [25,31,46]. Nonetheless, in this study, the increase was estimated to be below 1000-fold because the trigger was an event such as parturition rather than an acute disease.

Further, HPT significantly decreases over time after calving, and various studies have used HPT as a postpartum disease predictor [30,37]. HPT levels tend to increase rapidly in dystocia, placental retention, and metritis, and are related to subsequent fertility and milk yield [30,37]. High HPT concentrations (over 100 μg/mL) after delivery are highly correlated with the periparturient disease occurrence, and are known to increase 10–100 times in inflammatory states [17,25,36,37]. Here, because none of the cows exhibited diseases such as clinical metritis or other calving-related inflammation, a slight increase in HPT was observed rather than a value exceeding 100 μg/mL [37]. These APPs are synthesized when the APR is stimulated by IL-1, IL-6, interferon gamma, and TNF-α [4,31,34,43]. Therefore, we analyzed IL-6, a cytokine that stimulates the APR; nonetheless, no significant difference was detected. IL-6 induces inflammation by activating T and B cells and initiating the APR, but it is also classified as an embryokine and has been identified in the placenta, amnion, and myometrium [3]. Because IL-6 increases in late pregnancy and tends to steadily decrease after parturition, this result likely reflects overlap between its cytokine role and the period during which its concentration changes as an embryokine [3,19,43]. Moreover, because these cytokines are mainly secreted by macrophages or neutrophils at the site of injury, they may not increase markedly unless there is severe injury or trauma after delivery [17,43].

No significant differences were observed in the WBC count, another inflammatory marker. This may reflect the sampling time points used in this study. Unlike HPT, which increases 2–3 days after calving and recovers by 2–4 weeks, previous studies have shown that the WBC count and the ratio of albumin to globulin peak immediately after delivery and decline within 2–3 days [36]. Here, the initial sampling occurred 4.9 ± 1.7 and 4.2 ± 2.7 days after delivery in the ER and LR groups, respectively; thus, the WBC count did not exhibit a rapid change. Furthermore, WBCs are known to increase in subacute or chronic inflammation but decrease in acute inflammatory situations [33]. Because parturition is considered an acute event rather than a chronic condition, the WBC counts did not significantly increase or decrease [36].

COR is used as a primary biomarker of stress or a parameter for measuring stress caused by pain or discomfort [32,33,40]. It is an endogenous glucocorticoid that increases under stress and affects the immune response [29,39,40]. Notably, blood COR levels primarily reflect recent stress, tend to increase slightly in parturients, and regulate female reproductive function [29,33,40]. Here, COR concentration was highest immediately after parturition and decreased thereafter, but there was no significant difference, potentially because COR is also affected by environmental stress factors, such as handling, restraint, sampling procedures, and changes in the herd [33]. Moreover, in dairy cows, the stress caused by these changes, due to significant changes in the herd, feeding, and environment during peripartum, may be greater than in other animals [33,40].

Although SP has primarily been used as a pain-specific marker, it has recently been studied as a stress marker [33,38,40,41,42]. In particular, it is used to assess nociception, namely the sense of pain itself—as well as memory and responses [32,33,38,42,47,48]. Accordingly, some studies have shown decreases in SP following castration or other treatments, depending on the use of nonsteroidal anti-inflammatory drugs [32,33,38,42,47,48]. Furthermore, SP tends to increase with transport stress and anxiety, and heat stress can produce significantly higher levels of SP and COR [33,39]. Although SP is involved in immune cell migration and chemokine expression and has been used as a marker of inflammation in several studies, it is not considered a sensitive major APP, such as HPT or SAA [42]. Here, SP appeared to decrease over time immediately after calving; however, because of differences in pain perception and temperament among individuals, substantial interindividual variability was observed, resulting in no significant differences or trends [47].

The results of the T-Chol analysis to evaluate the recovery of feed intake in postpartum cows demonstrated significantly higher values in periods 2 and 3 than they did in period 1 (*p* < 0.001); specifically, the LR group exhibited significantly higher T-Chol levels in period 3 than it did in period 2 (*p* < 0.01). Moreover, β-HB increased within the normal range after parturition and then showed a tendency to decrease, whereas NEFA decreased significantly as time passed after parturition (*p* < 0.05). Because the NEB state persists for a certain period from the start of lactation after parturition until feed intake sufficiently recovers, β-HB appears to have increased slightly. However, when interpreted together with other values, such as T-Chol, it can be inferred that NEB resolved after a certain period.

Here, we analyzed several hematological parameters according to the recovery rate in postpartum cows. Nevertheless, due to the 1-week interval between examinations and group separation based on clinical uterine recovery, accurately determining uterine recovery was challenging. Although uterine recovery assessment by a skilled veterinarian may be meaningful based on the results of this study, such subjective and empirical judgment has limitations in general application. Nevertheless, significant differences were identified in SAA, HPT, T-Chol, and NEFA levels. Specifically, SAA and HPT levels were significantly lower after recovery than immediately post-parturition (*p* < 0.001). Consistent with previous studies, this suggests that HPT and SAA are more sensitive biomarkers of inflammation than the other parameters [2,28]. Furthermore, T-Chol and NEFA can be used as indicators of energy balance. In the field, VWP for dairy cows has been established based on uterine evaluations, such as rectal examinations, and the reproductive goals of traditional dairy farms, rather than on hematological analyses. Moreover, in many practical situations, estrus synchronization protocols are initiated without prior hematological assessment, which may overlook valuable information regarding the metabolic and physiological status of the animals [49]. However, customizing individual VWP based on HPT and SAA, rather than the existing goal VWP, can shorten unnecessary days open and improve conception rates by reducing the number of inseminations required for conception. Furthermore, allowing each cow sufficient recovery time could improve reproductive efficiency and reduce economic losses for the farm. While this study identified biomarkers that could be used as indicators among various parameters, further research is needed to continuously collect postpartum data and establish cut-off values for SAA and HPT by parity, which reflect postpartum recovery. Furthermore, through an examination of the farm size at which individual VWP settings are feasible, ultimately, a VWP decision model can be developed.

## 5. Conclusions

Determining the degree of uterine recovery through a clinical examination requires individual skills and can be subjective. Nevertheless, postpartum recovery in cows could be assessed objectively using the APP and metabolic markers identified in this study. This allows for the individual assessment of uterine recovery through blood analyses performed approximately 30 days after parturition, enabling the establishment of individual VWPs. Therefore, the development of hematological markers, including SAA and HPT, could contribute to improved productivity by enhancing the reproductive performance of farms. To achieve this, further research is warranted to establish cutoff values for each marker in different parities.

## Figures and Tables

**Table 1:** Information about each group.

Group	ER		LR	
**Recovery day after calving**	30.3 ± 1.5		51.5 ± 2.7	
**Parity**	1.5 ± 0.2		1.7 ± 0.2	
**Analysis point** **(day after calving** **)**	ER1 (period 1)	4.9 ± 0.5	LR1 (period 1)	4.2 ± 0.8
	ER2 (period 2)	30.3 ± 1.5	LR2 (period 2)	29.1 ± 1.1
			LR3 (period 3)	51.5 ± 2.7

All values are expressed as mean ± standard error. ER, early recovery; LR, late recovery.

## Data Availability

The original contributions presented in this study are included in the article/Supplementary Material. Further inquiries can be directed to the corresponding authors.

## Supplementary Materials

The following supporting information can be downloaded at: https://www.mdpi.com/article/10.3390/ani15223331/s1, Table S1: Results of all measured parameters.

## Abbreviations

The following abbreviations are used in this manuscript:

AI	Artificial insemination
APP	Acute phase protein
APR	Acute phase reaction
β-HB	Beta-hydroxybutyrate
COR	Cortisol
CRP	C-reactive protein
EDTA	Ethylenediaminetetraacetic acid
ELISA	Enzyme-linked immunosorbent assay
ER	Early recovery
HPT	Haptoglobin
IL	Interleukin
LR	Late recovery
NEB	Negative energy balance
NEFA	Non-esterified fatty acid
SAA	Serum amyloid A
SP	Substance P
T-Chol	Total cholesterol
TNF-α	Tumor necrosis factor-alpha
VWP	Voluntary waiting period
WBC	White blood cell (count)
