# Supplementary material for: Pilot Study: Exploring the Feasibility of Individual Voluntary Waiting Period Settings Using Postpartum Recovery Indicators in Dairy Cows
Source: Animals (Basel). 2025 Nov 19;15(22):3331. doi: 10.3390/ani15223331 (PMC12649167; doi:10.3390/ani15223331)
Supplement: Supplementary file 1 [file animals-15-03331-s001.zip › animals-3960183-supplementary.pdf]

animals

MDPI

Supplementary Table S1. Results of all measured parameters.

	Period 1		Period 2		Period 3
	ER	LR	ER	LR	LR
WBC (10^3/mm^3)	9.36±0.69	8.26±0.76	8.32±0.52	7.32±0.48	7.75±0.61
SAA (μg/mL)	115.03±14.65	105.81±16.71	37.68±8.47***	48.71±14.35*	28.53±10.39**
HPT (μg/mL)	27.27±7.06	22.77±6.87	2.35±1.61**	6.27±5.04	1.78±1.37*
COR (ng/mL)	36.47±10.49	29.59±7.51	31.10±12.45	25.15±4.57	39.04±14.55
SP (pg/mL)	4510.04±428.20	4053.68±669.45	3673.60±317.76	3746.08±380.42	4140.8±498.92
IL-6 (ng/mL)	12.68±0.63	12.41±0.33	12.15±0.30	12.35±0.28	12.18±0.17
T-Chol (mg/dL)	74.9±7.00	79.4±8.22	181.6±16.74***	154.8±19.09***,a	211.6±14.18***,b
β-HB (mmol/L)	0.55±0.06	0.61±0.07	0.71±0.11	0.99±0.18	0.88±0.18
NEFA (mmol/L)	0.42±0.10	0.44±0.10[a]	0.25±0.04	0.25±0.06	0.24±0.05[b]

* Significant differences are denoted (*, $p < 0.05$; **, $p < 0.01$; ***, $p < 0.001$; a-b, $p < 0.01$). ER, early recovery; LR, late recovery; WBC, white blood cell count; SAA, serum amyloid A; HPT, haptoglobin; COR, cortisol; ; SP, substance P; IL-6, interleukin-6; T-Chol, total cholesterol; β-HB, beta-hydroxybutyrate; NEFA, non-esterified fatty acids.
